# Marine Cyclotripeptide X-13 Promotes Angiogenesis in Zebrafish and Human Endothelial Cells via PI3K/Akt/eNOS Signaling Pathways

**DOI:** 10.3390/md10061307

**Published:** 2012-06-07

**Authors:** Xi-Lin Lu, Zhong-Liang Xu, Xiao-Li Yao, Feng-Juan Su, Cheng-Hui Ye, Jing Li, Yong-Cheng Lin, Guang-Lei Wang, Jin-Sheng Zeng, Ru-Xun Huang, Jing-Song Ou, Hong-Shuo Sun, Li-Ping Wang, Ji-Yan Pang, Zhong Pei

**Affiliations:** 1 Department of Neurology, The First Affiliated Hospital, Sun Yat-sen University, Guangzhou 510080, China; Email: luxilin@mail.sysu.edu.cn (X.-L.L.); yeyaoxiaoli@sohu.com (X.-L.Y.); jisufengjuan@163.com (F.-J.S.); ych201176@163.com (C.-H.Y.); zengjs@pub.guangzhou.gd.cn (J.-S.Z.); hrx998@yahoo.com.cn (R.-X.H.); 2 School of Chemistry & Chemical Engineering, Sun Yat-sen University, Guangzhou 510275, China; Email: xzhliang1979@163.com (Z.-L.X.); zsulijing@163.com (J.L.); ceslyc@mail.sysu.edu.cn (Y.-C.L.); 3 Department of Pharmacology, Zhongshan School of Medicine, Sun Yat-sen University, Guangzhou 510080, China; Email: wangglei@mail.sysu.edu.cn; 4 Key Laboratory of Functional Molecules from Oceanic Microorganisms, Department of Education of Guangdong Province, Guangzhou 510080, China; 5 Division of Cardiac Surgery, The First Affiliated Hospital, Sun Yat-sen University, Guangzhou 510080, China; Email: oujs@mail.sysu.edu.cn; 6 The Key Laboratory of Assisted Circulation, Ministry of Health, Guangzhou 510182, China; 7 Division of Anatomy, Department of Surgery, Institute of Medical Science, Faculty of Medicine, University of Toronto, Toronto, ON M5G 1G6, Canada; Email: hss.sun@utoronto.ca; 8 Shenzhen Institutes of Advanced Technology, Chinese Academy of Sciences, Shenzhen 518055, China; Email: lp.wang@siat.ac.cn

**Keywords:** angiogenesis, marine cyclotripeptide, zebrafish, endothelial nitric oxide synthase, xyloallenoide A

## Abstract

Cyclotripeptide X-13 is a core of novel marine compound xyloallenoide A isolated from mangrove fungus *Xylaria* sp. (no. 2508). We found that X-13 dose-dependently induced angiogenesis in zebrafish embryos and in human endothelial cells, which was accompanied by increased phosphorylation of eNOS and Akt and NO release. Inhibition of PI3K/Akt/eNOS by LY294002 or L-NAME suppressed X-13-induced angiogenesis. The present work demonstrates that X-13 promotes angiogenesis via PI3K/Akt/eNOS pathways.

## Abbreviations

PI3K: phosphatidylinositol 3-kinase
Akt: alpha serine/threonine-protein kinase
eNOS: endothelial nitric oxide synthase
HUVEC: human umbilical vein endothelial cells

## 1. Introduction

Ischemic cardiac and cerebrovascular diseases are the leading causes of death and adult disability in the world. In addition, many ischemic diseases such as limb ischemia also severely affect the daily life of patients. Restoration of blood supply to the ischemic tissues and organs is the most critical aspect of treatment of ischemic diseases.

Angiogenesis is the process of sprouting new blood vessels from existing blood vessels and plays a pivotal role in reestablishment of blood flow to the ischemic organs to overcome the ischemic insult. Thus, stimulation of angiogenesis represents an exciting approach for the treatment of ischemic disorders and the clinical translation of novel angiogenic agents will benefit millions of patients suffering from those disorders. 

Zebrafish embryo is well suited to the study of blood vessel formation because the vascular system has been well described in the developing zebrafish embryo and many pathways involved in angiogenesis in mammals are highly conserved in the zebrafish. Recently, several transgenic zebrafish lines with fluorescent blood vessels have been developed and these transgenic animals have been used successfully in the field of angiogenesis drug screening [1]. Compounds can directly add to the fish culture media, diffuse into the embryo and induce observable angiogenic effects.

**Chart 1:**
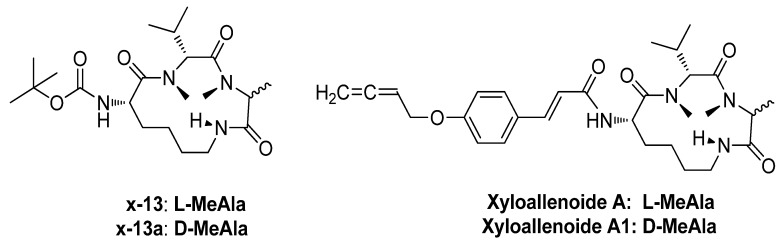
Structures of X-13 and Xyloallenoide A and its Derivatives.

The marine environment is a rich source of novel and unusual secondary metabolites for drug discovery. Cyclotripeptide X-13 is a core of novel marine compound xyloallenoide A (Chart 1), which was isolated from mangrove fungus *Xylaria* sp. (no. 2508) in the South China Sea [2]. Previously, we have demonstrated the total synthesis of xyloallenoide A and its diastereomer xyloallenoide A1 [3]. To explore their potential biological functions, we investigated whether these compounds have vascular activity. In the present study, we first demonstrated that X-13 and its derivatives concentration-dependently promoted the angiogenesis in zebrafish *in vivo* and endothelial cell cultures *in vitro*. The mechanism of angiogenic action of X-13 was also investigated in endothelial cell cultures. X-13 induced phosphorylation of eNOS and Akt in parallel with angiogenesis. Consistently, PI3K inhibitor or NOS inhibitor but not ERK1/2 inhibitor significantly reduced X-13-induced phosphorylation of eNOS and Akt and angiogenesis. Thus, our data suggest that X-13 induces angiogenesis through PI3K/Akt/eNOS pathway.

## 2. Results and Discussion

### 2.1. The Synthesis of Marine Cyclotripeptide X-13 and Xyloallenoide A

**Scheme 1:**
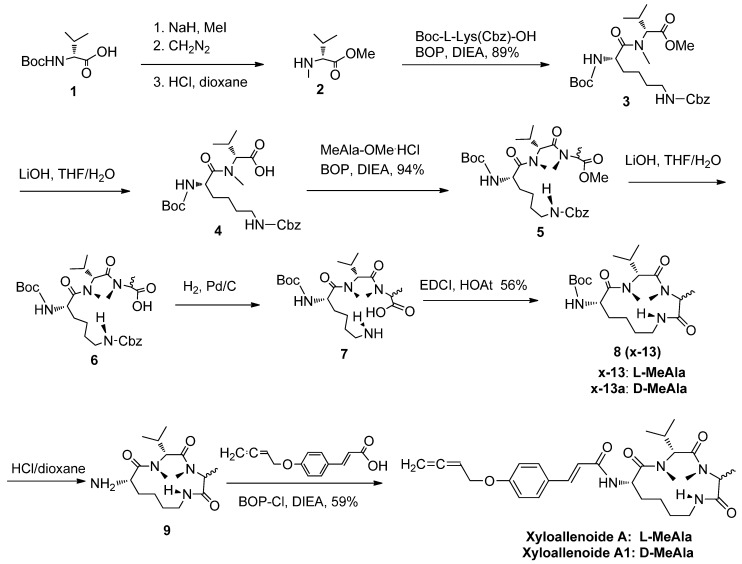
Synthesis of X-13 and xyloallenoide A and derivatives.

All the tested compounds were synthesized according to the literature (Scheme 1) [3]. Generally, Boc-L-Lys(Cbz)-OH, Boc-D-Val-OH, L-MeAla-OMe and D-MeAla-OMe were used as starting materials and BOP, EDCI, and BOP-Cl as coupling reagents. The *N*-methylation of Boc-D-Val-OH (**1**) was performed with MeI/NaH in THF at room temperature, followed by esterification and deprotection to form D-MeVal-OMe (**2**). Dipeptide **3 **was obtained by coupling **2 **and Boc-L-Lys(Cbz)-OH in 89% yield, which was further subjected to saponification to provide **4** in 87% yield. The tripeptide **5** was prepared by coupling dipeptide **4** with L-MeAla-OMe or D-MeAla-OMe in a good yield. The tripeptide **5 **was then saponificated using LiOH in THF/H_2_O, followed by removal of the Cbz group with 94% overall yield in two steps to afford the cyclizing precursor **7**. The cyclpeptide **8** was smoothly produced with EDCI/HOAt in moderate 56% yield. Final deprotection of the Boc group in **8** gave **9** in 96% yield. The xyloallenoide A and A1 was obtained by coupling of cyclictripeptide with 3-(4-buta-2,3-dienyloxy-phenyl)-acrylic acid [4] using BOP-Cl as catalyst in an acceptable 59% yield. All the target molecules were purified through a flash column chromatography.

### 2.2. Angiogenic Activity in Zebrafish Angiogenesis Screen

**Figure 1:**
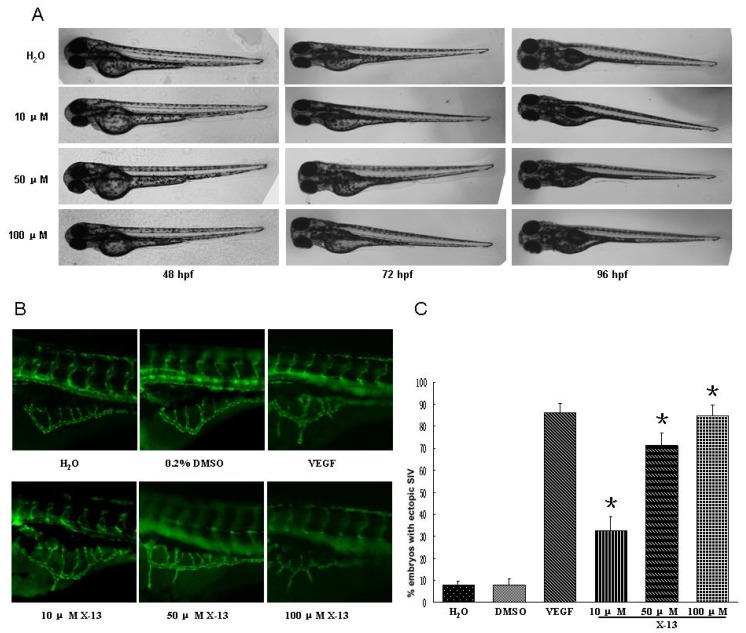
Effects of X-13 on angiogenesis in TG (Fli-1:EGFP) zebrafish embryos. (**A**) Representative brightfield images of zebrafish larvae from 48 to 96 hpf. Treatment with X-13 for up to 96 h did not adversely affect the normal development of zebrafish larvae; (**B**) Representative fluorescence microscopy images; and (**C**) The bar chart shows quantitative data. X-13 (10, 50, 100 μM) dose-dependently induced angiogenesis in zebrafish embryos with EGFP expressing SIV. The angiogenic activity of X-13 at 50 μM and beyond was comparable to that of VEGF at 100 ng/mL. Data are expressed as the mean ± SD. Results were obtained from six independent experiments (* *p* < 0.05 *vs*. control vehicle group).

Following synthesis of Cyclotripeptide X-13, their angiogenic action was examined in transgenic zebrafish with fluorescent blood vessels (Figure 1). When zebrafish embryos were incubated with tested compounds at final concentration 100 μM for 72 h, all compounds induced development of ectopic subintestinal vessels (SIV) in zebrafish embryos as evidenced by longer and more numerous spikes projecting from the subintestinal vessel basket, suggesting that all examined compounds had angiogenic activity. The percentage of ectopic SIV was 7.8 ± 1.5% at control, 71.2 ± 5.9% at X-13-treated group, 74.5 ± 6.4% at X-13a-treated group, 22.5 ± 3.6% at xyloallenoide A-treated group and 28.3 ± 4.2% at xyloallenoide A1-treated group, respectively. Cyclotripeptide X-13 and X-13a had stronger angiogenic action compared with nature marine product xyloallenoide A and synthesized xyloallenoide A1. It should be noted that the steric hinerance of substituted allenic cinnamate of xyloallenoide A and A1 at lysine residue was unfavorable for the activity. Furthermore, the difference in angiogenic action was very subtle between compound X-13 and X-13a or between xyloallenoide A and xyloallenoide A1, suggesting that absolute stereochemistry of alanine in the cyclotripeptide core is not essential for their angiogenic action. Given that X-13 and X-13a had similar biological activity, the nature core cyclotripeptide X-13 was used in the further bioactivity evaluation. In the dose response experiments, cyclotripeptide X-13 at 10, 50 and 100 μM dose-dependently increased the number of newly formed vessels and the plateau concentration was 50 μM (Figure 1). The angiogenic activity of X-13 at 50 μM and beyond was comparable to that of VEGF at 100 ng/mL. Therefore, a dose of 50 μM X-13 was used for subsequent experiments. 

### 2.3. *In Vitro* Angiogenesis in Mammalian Endothelial Cells

**Figure 2:**
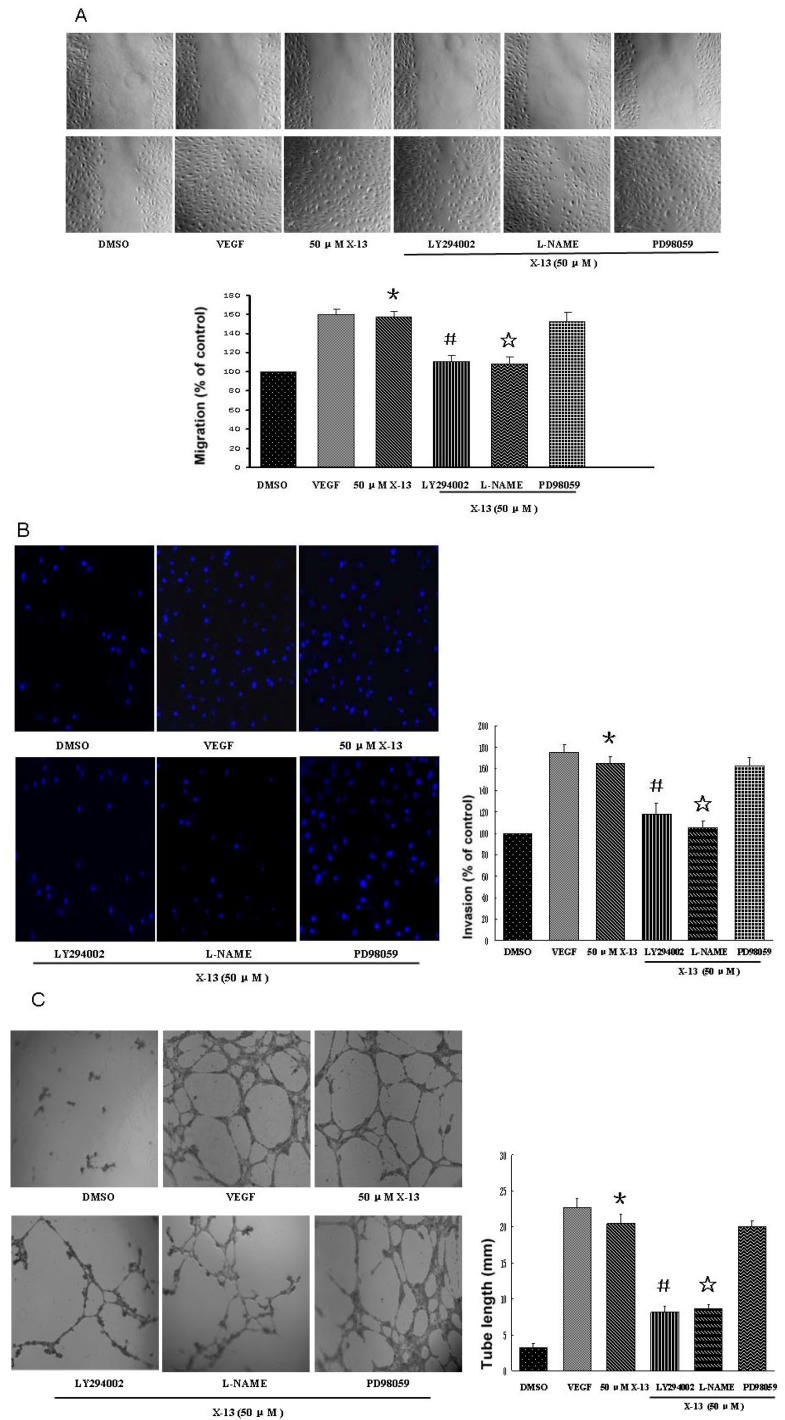
The effects of X-13 on Human Umbilical Vein Endothelial Cell (HUVEC) invasion, migration and tube formation. HUVEC cultures were incubated with X-13 in the presence or absence of inhibitors for different periods in different assays and VEGF-treated cell cultures served as a positive control. Representative images show that X-13 induced HUVEC migration (**A**), invasion (**B**) and tube formation (**C**) in HUVEC cultures. L-NAME and LY294002 significantly inhibited X-13-induced cell invasion (**A**), migration (**B**) and tube formation (**C**). The bar chart shows quantitative data. Data are expressed as the mean ± SD. Results were obtained from four independent experiments (* X-13 *vs*. vehicle control group, *p* < 0.05; # LY294002 plus X-13 *vs*. X-13, *p* < 0.05; ☆ L-NAME plus X-13 *vs*. X-13, *p* < 0.05).

Endothelial cells play a pivotal role in angiogenesis and activation of endothelial cells is the first step in angiogenesis. To form new blood vessels, endothelial cells must go though basement membrane invasion, cell migration, cell proliferation, and tube formation, of which every process can be a target for intervention [5]. To investigate whether our zebrafish findings can be translated into human studies and to explore the mechanisms responsible for X-13-induced angiogenesis, the biological action of X-13 was studied in Human Umbilical Vein Endothelial Cell (HUVEC) cultures. PI3K/Akt/eNOS and ERK1/2 are two major pathways in endothelial angiogenesis. Therefore, the inhibitors for PI3K (LY294002), eNOS (L-NAME) and ERK1/2 (PD98059) were used to explore the potential molecular pathways underlying X-13-induced angiogenesis. We first examined the effect of X-13 on HUVEC migration using the wound-healing method. There was no significant HUVECEC migration in vehicle control-treated HUVECs at 12 h post-wounding. In contrast, X-13 treatment significantly increased HUVEC migration (Figure 2A). L-NAME or LY294002 but not PD98059 significantly inhibited X-13-induced increase in HUVEC migration (Figure 2A). We then examined whether X-13 induces endothelial cell invasion using transwell culture inserts. Compared with the vehicle controls, there was a significant increase in the invasion of HUVECs treated with X-13. Treatment with L-NAME or LY294002 but not PD98059 significantly attenuated X-13-induced enhancement of HUVEC invasion (Figure 2B).

We further examined the effect of X-13 on HUVEC tube structure formation using Matrigel tube formation assay. Treatment of HUVECs with X-13 induced an extensive formation of capillary-like structures in a dose-dependent manner (Figure 2C). The increase in HUVEC tube formation was comparable between X-13-treated (50 μM) and VEGF-treated (100 ng/mL) groups. Furthermore, L-NAME or LY294002 but not PD98059 inhibited X-13-induced HUVEC tube formation (Figure 2C). 

Consistent with our zebrafish findings, we found that X-13 treatment increased endothelial cell invasion and augmented the migration of HUVECs, leading to a significant increase in HUVEC tube formation, a hallmark feature of angiogenesis in endothelial cells. 

### 2.4. PI3K/Akt/eNOS Activities in HUVECs

**Figure 3:**
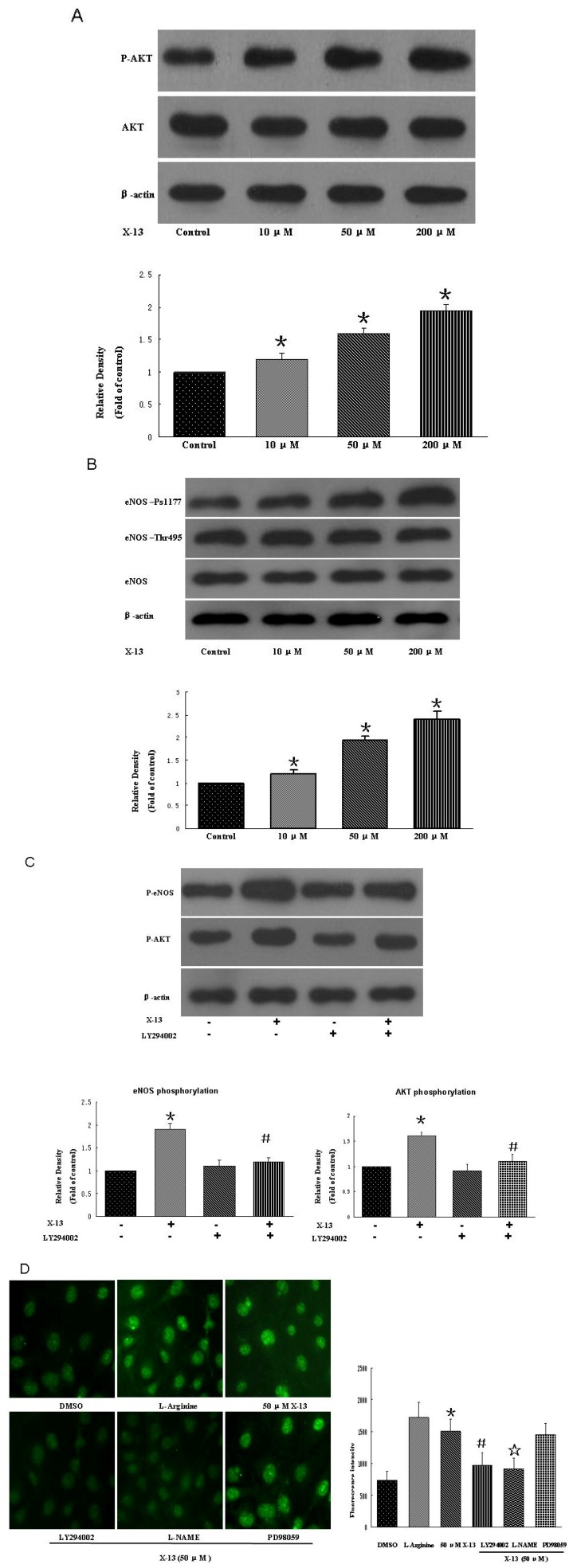
Activation of Akt/PI3K pathways and nitric oxide (NO) production in X-13-treated HUVECs. Representative blots show that X-13 increased Akt and eNOS phosphorylation (**A**,**B**) while PI3K inhibitor LY294002 attenuated X-13-induced Akt and eNOS phosphorylation (**C**). Representative image shows that X-13 induced NO release while PI3K inhibitor LY294002, NOS inhibitor LAME, but not erk1/2 inhibitor PD98059 reduced X-13-induced NO release (**D**). The bar charts show quantitative data. Data are expressed as the mean ± SD. Results were obtained from three independent experiments (* X-13 *vs*. vehicle control group, *p* < 0.05; # LY294002 plus X-13 *vs*. X-13, *p* < 0.05; ☆ L-NAME plus X-13 *vs*. X-13, *p* < 0.05).

Understanding compound-induced signal pathway is essential for development of safe and effective drugs. In previous experiments, L-NAME or LY294002 but not PD98059 inhibited X-13-induced angiogenesis tube formation, suggesting that eNOS and PI3K pathways might be involved in X-13-stimulated angiogenesis. To precisely investigate the signal transduction mechanisms responsible for X-13-induced angiogenesis, we examined the role of PI3K/Akt/eNOS pathway in X-13-induced angiogenesis. It is generally believed that endothelium-derived nitric oxide (NO) is a critical mediator of angiogenesis. eNOS catalyzes the synthesis of NO in blood vessels and thus has an important role in angiogenesis. It has been well documented that PI3K and its downstream effector Akt are implicated in the activation of eNOS. When Akt is activated following PI3K stimulation, it phosphorylates eNOS and the latter enhances NO release, thereby promoting angiogenesis [6]. In the present study, HUVECs were treated with different concentrations of X-13 and phosphorylation and expression of eNOS and Akt were examined by western blot (Figure 3). We found that X-13 dose-dependently increased Akt phosphorylation at site 473 and eNOS phosphorylation at site 1177 without altering the expression of total eNOS and Akt (Figure 3A,B). In contrast, X-13-induced Akt and eNOS phosphorylation was abolished in the presence of PI3K inhibitor LY294002 (Figure 3C). In addition, X-13 significantly increased NO generation while pre-incubation of HUVECs with L-NAME or LY294002 but not PD98059 dramatically inhibited X-13-induced NO generation (Figure 3D). Together, these findings suggest that PI3K/Akt/eNOS is responsible for the angiogenic action of X-13. 

## 3. Experimental Section

### 3.1. Preparation of Reagents and Cell Culture

All reagents and solvents were of commercial quality and used without further purification. ^1^H and ^13^C NMR data were recorded on a Varian Inova 400 MB NMR spectrometer operating at 400 and 100 MHz for ^1^H and ^13^C respectively. All chemical shifts are in ppm (*δ*) with respect to tetramethylsilane (TMS) as internal standard, and coupling constants (*J*) are in Hz. Mass Spectra on DSQ (Low Resolution Mass Spectrometer) and MAT95XP (High Resolution Mass Spectrometer). Melting point was determined on X-4 micro-melting point apparatus and was uncorrected. The purities (>95%) of all target compounds were checked by HPLC using a LC-2010c equipped with UV detector. Samples were injected on a Merck Purospher STAR RP-18e 125 cm × 4.6 mm (5 μm) column equipped with a Merck Lichrocart precolumn (Merck, Darmstadt, Germany). HUVECs were obtained from Sciencell, USA. HUVECs (Passage 4 to 7) were cultured at 37 °C in M199 media (Invitrogen, Carlsbad, NM, USA) supplemented with 10% FBS. Cells were maintained in a humidified atmosphere of 5% CO_2_. The angiogenic effect of X-13 on HUVECs was evaluated using a stock solution of X-13 (100 mg/mL) prepared in PBS containing 10% DMSO (GIBCO, Langley, VA, USA). The angiogenic effect of X-13 on zebrafish was evaluated using a stock solution of X-13 (1 mg/mL) prepared in sterilized Milli-Q water containing 0.5% DMSO. Vascular endothelial growth factor (VEGF) was obtained from Sigma, St. Louis, MO, USA and prepared as a stock solution of 100 μg/mL in sterilized Milli-Q water.

### 3.2. Angiogenesis in Zebrafish Embryos

The transgenic zebrafish cell line TG (fli1: EGFP), in which endothelial cells express eGFP, was kindly provided by ZFIN (Eugene, OR, USA) and maintained as described [7]. Zebrafish embryos were generated by natural pair-wise mating of fish that were between 3 and 12 months old. Embryos were collected as described. Healthy embryos were harvested at the 1–4 cell stage, plated in a 24-well microplate and treated with different compounds. Embryos receiving DMSO (0.2%) served as a vehicle control. VEGF, serving as positive control, was injected either into the yolk ball or into the perivitelline space between the yolk and periderm. Embryos were incubated at 28 °C for 72 h and were then anesthetized using 0.05% 2-phenoxyethanol in embryo water. One to three embryos were placed in each well of a 96-well plate and pro-angiogenic effects were evaluated by examining each embryo for the presence of ectopic vessels in the SIV as previously described [8]. The criteria for angiogenesis includes: (a) presence of vessels spiking out of the basket structure; and/or (b) extension of the SIV basket into the yolk extension region with more than seven vertical branches within the basket [8]. Thirty embryos in each experiment in each group were calculated.

### 3.3. Cell Invasion, Migration and Tube Formation

HUVEC invasion was investigated as described [9]. Briefly, the effect of X-13 on HUVEC invasion was measured using the 10 mm tissue culture insert (transwell) with polycarbonate membrane (8 mm pores) and 24-well companion plate. The upper side and lower side of the membrane were pre-coated with 1:30 (v/v) and 1:100 (v/v) of Matrigel, respectively. The HUVECs were resuspended in low serum (1% FBS) medium and seeded onto the culture inserts at 5 × 10^4^ cells per insert in triplicate. They were then deposited into the 24-well companion plate with 500 μL of low serum (1% FBS) medium containing X-13 (50 μM) in the presence or absence of LY294002 (10 μM), L-NAME (100 μM) or PD98059 (50 μM). In addition, the wells of the companion plate, containing DMSO (0.1%) and 100 ng/mL VEGF, served as a vehicle control and positive control, respectively. The inserts were removed after 8 h of incubation and were then washed with PBS. Non-invasive cells on the upper surface of the membrane were removed by wiping with cotton swabs. The inserts were fixed in paraformaldehyate, stained with DAPI and mounted on microscope slides. Images of the invasive cells were captured at 100× magnification using a fluorescent inverted microscope and a CCD camera. Following this, HUVEC invasion was quantified by counting the number of cells per insert with the software Metamorph Imaging Series (Molecular Devices, Tokyo, Japan).

HUVEC migration assay was performed using the wound healing method as previously described [10]. The HUVECs (3 × 10^5^ cells) were seeded into each well of a 24-well plate and incubated with complete medium at 37 °C and 5% CO_2_. After 24 h of incubation, cells were starved for additional 24 h by low serum (0.5% FBS) medium. The HUVECs were then scraped away horizontally in each well using a P100 pipette tip. Three randomly selected views along the scraped line were photographed on each well using a fluorescent inverted microscope and the CCD camera attached to the microscope at 50× magnification. The medium was then changed to fresh low serum (1% FBS) medium with or without X-13 (50 μM) in the presence or absence of LY294002 (10 μM), L-NAME (100 μM) or PD98059 (50 μM). After 12 h of incubation, another set of images were taken by the same method. Image analysis for signs of migration was performed by Metamorph Imaging Series. The average scraped area of each well under each condition was measured and subtracted from that of the before-treatment condition. Data are expressed as percentage wound closure relative to the wound closure area in the control medium. The wound closure area of the control cells was set at 100%.

Endothelial tube formation was assessed in 24-well plates using growth factor-reduced Matrigel™ as described previously [11,12]. Briefly, growth factor-reduced Matrigel (250 μL) was pipetted onto 24-well culture plates and polymerized for 30 min at 37 °C. HUVECs were seeded on Matrigel-coated plates at a density of 5 × 10^4^ in low serum (1% FBS) medium containing X-13 together with or without LY294002, L-NAME or PD98059 and then incubated at 37 °C for 8 h. Cells receiving DMSO (0.1%) served as a vehicle control and were equivalent to no treatment. In addition, cells cultured in 100 ng/mL VEGF served as a positive control. The network-like structures were examined under an inverted microscope (at 50× magnification). Tube-like structures were defined as endothelial cord formations that were connected at both ends. The tube length was quantified using the software NIH Image as reported earlier [11,12].

### 3.4. Western Immunoblot Analysis

HUVECs were plated in 60 mm dishes (16,700 cells/cm^2^) and cultured for 2 days. Culture medium was then replaced with fresh medium containing 2% FBS and the cells were treated with different concentrations of X-13 (10, 50, 100 μM) for 24 h. Cells were pretreated with or without LY294002 (10 μM) 30 min before exposure to X-13. Cellular proteins were harvested and immunoblots were performed as described [13]. The primary antibodies used were: antibody to anti-eNOS (Santa Cruz Biotechnology, Santa Cruz, CA, USA), anti-phosphorylation of eNOS at S1177 (P-eNOS S1177) (Cell Signaling Technology, Danvers, MA, USA), anti-phospho-Akt (Cell Signaling Technology, USA), anti-Akt (Cell Signaling Technology, USA), and anti-β-actin (Biosynthesis Biotechnology, Beijing, China). 

### 3.5. Measurement of NO Generation in HUVECs

NO generation was measured using a fluorescence method [14]. In this assay, 3-amino,4-aminomethyl-2′,7′-difluorescein, diacetate (DAF-FM-DA; Beyotime, Shanghai, China) was used as a fluorescent indicator of intracellular NO [14]. Briefly, HUVECs were seeded in 35-mm Petri dishes with a glass-bottom insert (MatTek, Ashland, MA, USA) at the density of 4 × 10^5^/well. After treatment with X-13 (50 μM) for 1 h, Cells were washed three times with colorless serum-free medium (GIBCO, Langley, VA, USA) and then incubated with 5 mmol/L DAF-FM-DA at 37 °C for 20 min. Cells were then rinsed three times to remove the excess probe and maintained in colorless serum-free medium throughout the experiments. DMSO (0.1%) and L-Arginine (1mM) were used as the vehicle control and positive control, respectively. LY294002 (10 μM), L-NAME (100 μM) or PD98059 (50 μM) were applied 10 min before X-13 treatment. Images were captured by an Olympus Fluoview laser confocal scanning microscope (IX81; Olympus, Tokyo, Japan) with a FITC parameter (excitation 494 nm, emission 518 nm). The fluorescence intensities were obtained using FV500 software (Olympus) to quantify the image of each group. 

### 3.6. Statistical Analysis

All data were presented as mean ± SEM. Differences among test groups were analyzed by ANOVA, using Newman-Keuls multiple comparison test (Prism 4.0, GraphPad Software, Inc., San Diego, CA, USA). A *p* value < 0.05 was considered statistically significant. 

## 4. Conclusions

Hundreds of thousands of patients suffer from ischemic vascular diseases and the fundamental problem underlying ischemic vascular diseases is the inability of tissue blood supply to match the tissue demand. Thus, therapeutic angiogenesis has a great promise for those with ischemic vascular diseases as therapeutic angiogenesis promotes angiogenesis and salvage ischemic tissues. 

In the current study, we have identified Cyclotripeptide X-13 as a series of novel marine angiogenic compounds. The PI3K/Akt/eNOS pathway is responsible for the angiogenic action of X-13 and the exact mechanism of X-13 action on the PI3K/Akt/eNOS pathway is currently under investigation. Given that X-13 is a small molecule with molecule weight under 1000 and possesses potent angiogenic property, it is very promising for development as a novel class of pro-angiogenic agents for angiotherapy.
